# Prevalence of and Recovery From Anemia Following Hospitalization for Critical Illness Among Adults

**DOI:** 10.1001/jamanetworkopen.2020.17843

**Published:** 2020-09-24

**Authors:** Matthew A. Warner, Andrew C. Hanson, Ryan D. Frank, Phillip J. Schulte, Ronald S. Go, Curtis B. Storlie, Daryl J. Kor

**Affiliations:** 1 Division of Critical Care Medicine, Department of Anesthesiology and Perioperative Medicine, Mayo Clinic, Rochester, Minnesota; 2 Division of Biomedical Statistics and Informatics, Department of Health Sciences Research, Mayo Clinic, Rochester, Minnesota; 3 Division of Hematology, Mayo Clinic, Rochester, Minnesota

## Abstract

**Importance:**

Anemia is common and has been associated with poor outcomes in the critically ill population, yet the timing and extent of hemoglobin recovery remains incompletely described, which may have important implications for clinical outcomes following discharge from intensive care.

**Objectives:**

To describe longitudinal changes in anemia status during and after critical illness and assess the associations between hemoglobin concentrations and postdischarge mortality.

**Design, Setting, and Participants:**

A population-based cohort study was conducted from January 1, 2010, to December 31, 2016, in Olmsted County, Minnesota; data analysis was performed from June 1 to December 30, 2019. Participants included 6901 adults (age ≥18 years) admitted to intensive care.

**Main Outcomes and Measures:**

Hemoglobin concentrations in the 12 months before hospitalization, during hospitalization, and in the 12 months after discharge, categorized by anemia severity (mild, hemoglobin ≥10.0 to <12.0 g/dL in women or ≥10.0 to <13.5 g/dL in men; moderate, hemoglobin ≥8.0 to <10.0 g/dL; and severe, hemoglobin <8.0 g/dL). Complete recovery from anemia, defined as attainment of nonanemic status by 12 months post hospitalization, and 12-month mortality were also evaluated.

**Results:**

Of the 6901 patients included in the study, 3792 were men (55%); median (interquartile range [IQR]) age was 67 (IQR, 52-79) years. Prehospitalization hemoglobin concentrations were available in 83% of the population (n = 5694), with median hemoglobin concentrations of 13.1 (IQR, 11.6-14.4) g/dL. Forty-one percent of the patients (n = 2320) had anemia preceding hospitalization. Hemoglobin values at hospital discharge were 10.8 g/dL (IQR, 9.5-12.4 g/dL), with 80% (n = 5182 of 6460) having anemia: 58% mild, 39% moderate, and 3% severe. The prevalence of anemia post hospitalization was 56% (95% CI, 55%-58%) at 3 months, 52% (95% CI, 50%-54%) at 6 months, and 45% (95% CI, 43%-47%) at 12 months among those alive with available hemoglobin measurements. Rates of complete recovery from anemia at 12 months were 58% (95% CI, 56%-61%) for mild anemia, 39% (95% CI, 36%-42%) for moderate anemia, and 24% (95% CI, 15%-34%) for severe anemia. Of those without baseline anemia surviving hospitalization, 74% of the patients were anemic at hospital discharge, with rates of complete 12-month recovery of 73% (95% CI, 69%-76%) for mild anemia, 62% (95% CI, 57%-68%) for moderate anemia, and 59% (95% CI, 35%-82%) for severe anemia. Higher hospital discharge hemoglobin concentrations were associated with decreased mortality after multivariable adjustment (hazard ratio, 0.95 per 1-g/dL increase; 95% CI, 0.90-0.99, *P* = .02).

**Conclusions and Relevance:**

The findings of this study suggest that anemia is common and often persistent in the first year after critical illness. Further studies are warranted to identify distinct anemia recovery profiles and assess associations with clinical outcomes.

## Key Points

**Question:**

Is anemia in patients with critical illness associated with persistent anemia following discharge?

**Findings:**

In this cohort study of 6901 adults hospitalized for critical illness, 41% had prevalent anemia preceding hospitalization and 74% of those without prehospitalization anemia developed incident anemia, for an overall anemia prevalence of 80% at hospital discharge. Rates of complete recovery from hospital discharge anemia at 12 months posthospitalization for those alive with available hemoglobin assessments were 58% for mild anemia, 39% for moderate anemia, and 24% for severe anemia.

**Meaning:**

The findings of this study suggest that anemia is common and often persistent in the first year after critical illness.

## Teaser

This cohort study examines the presence of anemia in critically ill patients and outcomes after discharge.

## Introduction

Anemia is a well-known complication of critical illness. Virtually all patients with intensive care unit (ICU) stays longer than 7 days experience anemia,^1^ and more than 75% of critical illness survivors are anemic at hospital discharge.^2^ Clinicians have generally become more tolerant of anemia given similar short-term mortality rates with restrictive vs liberal red blood cell (RBC) transfusion strategies.^3,4,5^ As such, the incidence of transfusion has decreased over time with a corresponding increase in the proportion of patients discharged with anemia.^6,7^ Consequences associated with anemia remain incompletely defined, although recent data suggest that anemia at ICU discharge is associated with reduced physical function 3 months later in critical illness survivors.^8^ Anemia has also been associated with reduced physical function and quality of life in noncritically ill populations.^9,10,11,12,13,14,15,16^

Despite the high prevalence of anemia during critical illness, longitudinal data profiling anemia development and subsequent recovery are lacking. While many critically ill patients are anemic at the time of ICU admission,^17,18,19^ it is unclear whether anemia develops abruptly during hospital admission or insidiously before hospitalization, which may have implications for the early identification of high-risk patients before the development of critical illness. Furthermore, data regarding the resolution or persistence of anemia beyond the initial hospital encounter are limited,^20^ representing a gap in our understanding of recovery from critical illness.

In this population-based investigation, we examined longitudinal changes in hemoglobin concentrations before, during, and after critical illness and assessed the associations between hospital discharge hemoglobin concentrations and posthospitalization mortality. Moreover, we assessed differences in anemia recovery based on the severity of anemia at hospital discharge, which is information that will be useful in facilitating future efforts assessing the associations between hemoglobin recovery and post-ICU outcomes.

## Methods

Residents of Olmsted County, Minnesota, were eligible for inclusion, with medical records obtained through the Rochester Epidemiology Project, a comprehensive epidemiologic database of population health information.^21,22^ Inclusion criteria included adult age (≥18 years), admission to an ICU during hospitalization from January 1, 2010, through December 31, 2016, and hemoglobin concentration available in the first 24 hours of hospitalization (ie, hospital admission hemoglobin concentration, defined as the first hemoglobin concentration measured during admission). For patients surviving critical illness, an independent hospital discharge hemoglobin concentration (ie, the last hemoglobin concentration measured before hospital discharge) must have been obtained in the 5 days before discharge. Survivors missing either of these measurements were excluded, as a principal study objective was to assess changes in hemoglobin concentrations during hospitalization and patients with only a single hemoglobin assessment would be unlikely representative of true critical illness. Only the first hospitalization with an ICU admission was included for each patient, such that no patients were included more than once.

This was a population-based cohort study conducted under institutional review board approvals from the Mayo Clinic and Olmsted Medical Center with waived requirement for written informed consent given minimal patient risk, although consistent with Minnesota statute 144.295 that patients who had previously denied authorization for medical record use in observational research were excluded. The Strengthening the Reporting of Observational Studies in Epidemiology (STROBE) guidelines were used in study design, conduct, and reporting of results.^23^

### Outcomes and Assessments

The primary outcomes were changes in hemoglobin concentrations and the prevalence of anemia at 3, 6, and 12 months after hospital discharge. Anemia was defined by hemoglobin values less than 12.0 g/dL for women or less than 13.5 g/dL for men (to convert to grams per liter, multiply by 10), with further classification by severity in accordance with previously used definitions^24,25^: mild (hemoglobin ≥10.0 g/dL and <12.0 g/dL for women or <13.5 g/dL for men), moderate (hemoglobin ≥8.0 and <10.0 g/dL), and severe (hemoglobin <8.0 g/dL). Additional outcomes included the prevalence of baseline anemia preceding hospitalization, identified using the most recent hemoglobin concentration measurement preceding hospital admission (within 12 months); the rate of incident hospital-acquired anemia; changes in hemoglobin concentrations from hospital admission to hospital discharge and ICU admission to ICU discharge; hemoglobin recovery or persistence in the first 12 months after hospital discharge, with complete recovery defined as attainment of nonanemic status; and 12-month posthospitalization all-cause mortality as ascertained through Minnesota Department of Health and National Death Index records.

The primary variables of interest were blood hemoglobin concentrations obtained in the 12 months before hospitalization, during hospitalization with associated critical illness, and in the 12 months after hospitalization. All hemoglobin measurements were extracted using the Rochester Epidemiology Project data repository, which includes all measurements obtained during inpatient, outpatient, and emergency health care encounters in southeastern Minnesota and western Wisconsin (27 counties).^22^ Hemoglobin concentration measurements were divided into predefined categories: prehospitalization (ie, in the 12 months before and up to 1 day before hospital admission, with the value closest to hospital admission used to define baseline hemoglobin concentration), hospital admission (ie, in the first 24 hours of hospitalization), ICU admission (ie, in the first 24 hours of ICU admission), ICU discharge (ie, in the 48 hours before ICU discharge; ICU discharge hemoglobin values could not be shared with ICU admission values), hospital discharge (the closest value to hospital discharge, occurring in the 5 days before discharge), and at 3, 6, and 12 months post hospitalization (within 2 months of each interval, without overlap such that any given hemoglobin value could be included only in the closest interval). Mean corpuscular volumes at hospital discharge were used to define anemia as microcytic (<80 μm^3^), normocytic (80-100 μm^3^), or macrocytic (>100 μm^3^) (to convert to femtoliters, multiply by 1). Additional exposures of interest included demographic characteristics (age, sex, and patient-reported race/ethnicity), baseline clinical characteristics (comorbidities and Charlson Comorbidity Index score, with minimum score of 0 [low severity of comorbid disease] and maximum score of 24 [high severity of comorbid disease]), hospitalization characteristics (ICU admission type [surgical vs nonsurgical]; ICU admission Sequential Organ Failure Assessment [SOFA] score, with minimum score of 0 [low organ failure severity] and maximum score of 24 [high organ failure severity], and Acute Physiology and Chronic Health Evaluation (APACHE) III score, with minimum score of 0 [low acute illness severity] and maximum score of 299 [high acute illness severity]; ICU length of stay; and hospital length of stay), transfusion characteristics (allogeneic RBC and non-RBC units transfused during hospitalization), and anemia management during hospitalization (RBC transfusions, iron, and erythropoiesis-stimulating agents).

### Statistical Analysis

Data analysis was conducted from June 1 to December 30, 2019. Patient demographic, laboratory, and admission characteristics were summarized as median (interquartile range [IQR]) for continuous variables and frequency (percent) for categorical variables according to prehospitalization anemia status. Anemia and mortality rates are summarized as number (percent). Given that hemoglobin measurements were not uniformly present in an observational database, not all patients had measurements available at each posthospitalization interval. In a primary approach, changes in hemoglobin concentration and anemia status with corresponding 95% CIs are reported for patients with available data (complete case analysis). Recognizing that missing hemoglobin data may be informative (eg, patient doing well without need for recheck, censored owing to death or change of residence, and transitioned to hospice care), we also provide comparative demographic and clinical characteristics by the number of posthospitalization hemoglobin concentration assessments (centered above and below the median value of 5: 0, 1-5, and >5). In addition, multiple imputation to account for missing values was conducted using 50 independent data sets and fully conditional specification, including age, sex, admission APACHE III score, admission type, hospital length of stay, log(follow-up time), last follow-up status (alive/dead), RBC transfusion during hospitalization, non-RBC transfusion during hospitalization, and available hemoglobin concentration values at hospital admission, ICU admission, hospital discharge, and 1, 3, 6, 9, and 12 months post hospitalization, with the values then converted to corresponding anemia status for those alive and not censored owing to change of residence. Assessments of anemia development and recovery are presented for the full cohort and subgrouped for surgical and nonsurgical patients (ie, no surgery during hospitalization). Multivariable Cox proportional hazards regression models were used to assess the associations between hospital discharge hemoglobin concentrations and instantaneous hazard of death in the first year post hospitalization. Potential confounding variables selected a priori and included in the model were age, sex, admission type, Charlson Comorbidity Index score, admission APACHE III score, ICU length of stay, and prehospitalization anemia status (none, mild, moderate, severe, and not available). Schoenfeld residuals were inspected visually to assess the assumption of proportional hazards. Hazard ratios (HRs), 95% CIs, and associated *P* values per 1-g/dL increase in hemoglobin value were reported. *P* values <.05 (2-sided) were considered statistically significant. All analyses were performed using SAS, version 9.4 (SAS Institute Inc).

## Results

A total of 6901 patients (3792 [55%] men; 3109 [45%] women) met inclusion criteria, without exclusions (eFigure 1 in the Supplement), with median (IQR) age of 67 (IQR, 52-79) years. Prehospitalization hemoglobin concentrations were available in 5694 patients (83%), with median time from measurement to hospitalization of 1.2 (IQR, 0.6-3.6) months. The median prehospitalization hemoglobin value was 13.1 (IQR, 11.6-14.4) g/dL; 2320 patients (41%) with available data had prevalent anemia before hospitalization. Those without prehospitalization hemoglobin concentration data and nonanemic patients were generally healthier than those with anemia, as evidenced by younger age and lower Charlson Comorbidity Index, SOFA, and APACHE III scores (Table 1). Median ICU length of stay was 1.2 (IQR, 0.8-2.2) days and median hospital length of stay was 4.8 (IQR, 2.8-7.8) days.

**Table 1.** Demographic and Clinical Features by Prehospitalization Anemia Status^a^

Category	No. (%)					
	Not available (n = 1207)	Not anemic (n = 3374)	Anemia			Overall (n = 6901)
			Mild (n = 1811)	Moderate (n = 422)	Severe (n = 87)	
**Patient characteristics**						
Age, median (IQR), y	57 (43-70)	66 (51-78)	74 (60-84)	72 (58-83)	67 (55-78)	67 (52-79)
Sex						
Women	413 (34)	1720 (51)	700 (39)	231 (55)	45 (52)	3109 (45)
Men	794 (66)	1654 (49)	1111 (61)	191 (45)	42 (48)	3792 (55)
Race/ethnicity						
White	1024 (85)	3059 (91)	1640 (91)	383 (91)	68 (78)	6174 (89)
African American	58 (5)	102 (3)	60 (3)	14 (3)	9 (10)	243 (4)
Asian	40 (3)	68 (2)	54 (3)	10 (2)	4 (5)	176 (3)
Other	85 (7)	145 (4)	57 (3)	15 (4)	6 (7)	308 (4)
Charlson Comorbidity Index score, median (IQR)	0 (0-1)	1 (0-3)	3 (1-6)	4 (2-7)	3 (1-5)	2 (0-4)
Myocardial infarction	42 (3)	294 (9)	273 (15)	62 (15)	6 (7)	677 (10)
Congestive heart failure	47 (4)	400 (12)	482 (27)	141 (33)	12 (14)	1082 (16)
Peripheral vascular disease	77 (6)	650 (19)	542 (30)	130 (31)	16 (18)	1415 (21)
Cerebrovascular disease	59 (5)	435 (13)	344 (19)	96 (23)	12 (14)	946 (14)
Dementia	23 (2)	150 (4)	136 (8)	37 (9)	3 (3)	349 (5)
Chronic pulmonary disease	172 (14)	1029 (30)	635 (35)	140 (33)	27 (31)	2003 (29)
Peptic ulcer disease	20 (2)	158 (5)	117 (6)	32 (8)	3 (3)	330 (5)
Diabetes	162 (13)	840 (25)	682 (38)	172 (41)	34 (39)	1890 (27)
Hemiplegia	13 (1)	82 (2)	47 (3)	9 (2)	2 (2)	153 (2)
Moderate/severe kidney disease	54 (4)	319 (9)	495 (27)	175 (41)	33 (38)	1076 (16)
Moderate/severe liver disease	4 (0)	31 (1)	31 (2)	5 (1)	1 (1)	72 (1)
Metastatic solid tumor	17 (1)	126 (4)	165 (9)	39 (9)	9 (10)	356 (5)
Rheumatologic disease	8 (1)	156 (5)	118 (7)	31 (7)	5 (6)	318 (5)
**ICU and hospital characteristics**						
ICU specialty						
Medical	373 (31)	1119 (33)	777 (43)	192 (45)	38 (44)	2499 (36)
Trauma/general surgical	259 (21)	484 (14)	207 (11)	42 (10)	11 (13)	1003 (15)
Neuroscience	128 (11)	315 (9)	94 (5)	11 (3)	6 (7)	554 (8)
Cardiac (medical)	297 (25)	435 (13)	227 (13)	40 (9)	4 (5)	1003 (15)
Vascular/thoracic/surgical	96 (8)	380 (11)	196 (11)	44 (10)	8 (9)	724 (10)
Medical/surgical/transplant	20 (2)	174 (5)	151 (8)	82 (19)	19 (22)	446 (6)
Cardiac (surgical)	34 (3)	467 (14)	159 (9)	11 (3)	1 (1)	672 (10)
Surgical admission^b^	391 (32)	1469 (44)	631 (35)	134 (32)	31 (36)	2656 (38)
Trauma admission	254 (21)	475 (14)	230 (13)	55 (13)	11 (13)	1025 (15)
APACHE III score, median (IQR)	47 (35-65)	53 (41-69)	64 (51-79)	69 (56-85)	68 (51-79)	56 (42-72)
SOFA score day 1 (n = 6883), median (IQR)	2 (1-5)	3 (1-6)	4 (2-6)	4 (2-7)	5 (3-7)	3 (1-6)
ICU length of stay, median (IQR), d	1.4 (0.9-2.5)	1.1 (0.8-2.0)	1.2 (0.8-2.2)	1.3 (0.9-2.4)	1.4 (0.9-2.2)	1.2 (0.8-2.2)
Hospital length of stay, median (IQR), d	3.9 (2.2-7.5)	4.6 (2.8-7.2)	5.3 (3.3-8.8)	6.0 (3.8-10.2)	5.7 (3.6-10.8)	4.8 (2.8-7.8)
Hospital mortality	50 (4)	170 (5)	169 (9)	47 (11)	5 (6)	441 (6)

Abbreviations: APACHE, Acute Physiology and Chronic Health Evaluation; ICU, intensive care unit; IQR, interquartile range; SOFA, Sequential Organ Failure Assessment.

^a^ Anemia status was defined according to hemoglobin values as nonanemic (≥13.5 g/dL in men; ≥12.0 g/dL in women) and mild (≥10.0 to <13.5 g/dL in men; ≥10.0 to <12.0 g/dL in women), moderate (≥8.0 to <10.0 mg/dL), and severe (<8.0 g/dL) anemia. To convert to grams per liter, multiply by 10.

^b^ Defined by admission to a surgical service or ICU admission immediately following a surgical procedure.

### Anemia During Hospitalization

**Figure 1.**
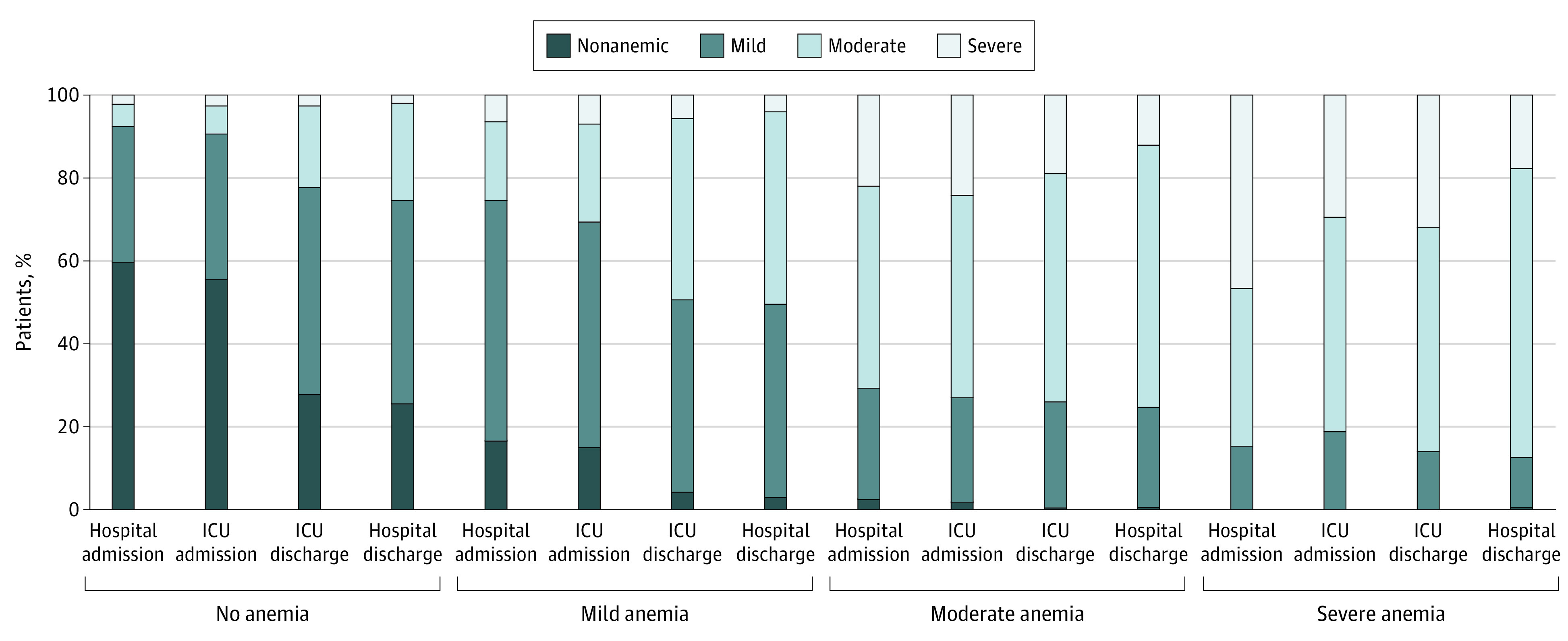
Changes in Anemia Status During Hospitalization Stratified by Prehospitalization Anemia Status ICU indicates intensive care unit.

The median change in hemoglobin from prehospitalization to hospital admission was −0.7 (IQR, −1.8 to 0.2) g/dL (Table 2). Of those who were previously nonanemic (n = 3374), 40% developed incident anemia within the first 24 hours. In total, 56% of patients were anemic within 24 hours of ICU admission, with this proportion increasing to 78% by ICU discharge, exclusive of 3% dying during ICU admission. The rate of incident anemia at ICU admission was 44% and the rate at ICU discharge was 71% for those with normal prehospitalization hemoglobin values. The median change in hemoglobin concentration from ICU admission to ICU discharge was −1.0 (IQR, −2.1 to −0.2) g/dL. The median change from prehospitalization to hospital discharge was −2.0 (IQR, −3.4 to −0.8) g/dL, with a median discharge hemoglobin concentration of 10.8 (IQR, 9.5-12.4) g/dL. At hospital discharge, the prevalence of anemia among survivors was 80% (n = 5182 of 6460: 58% mild, 39% moderate, and 3% severe). Longitudinal changes in anemia status by prehospitalization anemia status are displayed in Figure 1 and eTable 1 in the Supplement and by type of admission (surgical vs nonsurgical) in eFigure 2 in the Supplement. Of those who were nonanemic before hospitalization and survived to hospital discharge (n = 3204), only 26% remained nonanemic (74% incident anemia; 66% mild, 32% moderate, and 2% severe).

**Table 2.** Hemoglobin and Anemia Categories for Critically Ill Patients Before and During Hospitalization^a^

Category	Prehospitalization (n = 5694)	Admission		Discharge	
		Hospital (n = 6901)	ICU (n = 6845)	ICU (n = 6809)	Hospital (n = 6901)
Hemoglobin, median (IQR), g/dL	13.1 (11.6-14.4)	12.5 (10.8-14.0)	12.4 (10.5-13.9)	11.0 (9.5-12.6)	10.8 (9.5-12.4)
Women	12.5 (11.3-13.7)	11.9 (10.4-13.3)	11.7 (10.1-13.1)	10.5 (9.3-11.8)	10.4 (9.4-11.7)
Men	13.8 (12.1-15.0)	13.1 (11.3-14.5)	13.0 (11.0-14.4)	11.4 (9.8-13.1)	11.2 (9.6-13.0)
Anemia status, No. (%)^b^					
Nonanemic	3374 (59)	3214 (47)	2983 (44)	1437 (21)	1278 (19)
Mild	1811 (32)	2558 (37)	2545 (37)	3026 (44)	2980 (43)
Moderate	422 (7)	806 (12)	964 (14)	1821 (27)	2021 (29)
Severe	87 (2)	323 (5)	353 (5)	293 (4)	181 (3)
Death^c^	NA	NA	NA	232 (3)	441 (6)

Abbreviations: ICU, intensive care unit; IQR, interquartile range; NA, not applicable.

SI conversion: To convert hemoglobin to grams per liter, multiply by 10.

^a^ Hospital and ICU discharge hemoglobin values were considered missing for patients who did not survive to discharge from the given environment. Data here are based on available hemoglobin values.

^b^ Anemia status was defined according to hemoglobin values as nonanemic (≥13.5 g/dL in men; ≥12.0 g/dL in women) and mild (≥10.0 to <13.5 g/dL in men; ≥10.0 to <12.0 g/dL in women), moderate (≥8.0 to <10.0 mg/dL), and severe (<8.0 g/dL) anemia.

^c^ Patients must have survived to ICU admission for study inclusion; hence, there are no patients in the death category through ICU admission.

Most (91%) anemias were normocytic (Table 3). The incidence of RBC transfusion increased in accordance with prehospitalization anemia severity: 17% for those without anemia, 36% for mild anemia, 66% for moderate anemia, and 78% for severe anemia. Iron was administered during hospitalization to 298 patients (4%) and erythropoiesis-stimulating agents were administered to 19 patients (0.3%). There were no substantial changes in median hospital discharge hemoglobin concentrations over time, although the prevalence of severe anemia increased steadily from 1.0% in 2010 to 5.2% in 2016 along with a corresponding decrease in mild anemia (from 50.4% to 44.7%) (eTable 2 in the Supplement).

**Table 3.** Hemoglobin and Transfusion Details During Index Hospitalization by Prehospitalization Anemia Status

Variable	No. (%)					
	Not available (n = 1207)	Not anemic (n = 3374)	Anemia^a^			Overall (n = 6901)
			Mild (n = 1811)	Moderate (n = 422)	Severe (n = 87)	
Hemoglobin, median (IQR), g/dL^a^						
Initial	13.8 (12.5-15.0)	13.2 (11.9-14.3)	11.2 (10.0-12.4)	9.2 (8.1-10.2)	8.1 (6.7-9.2)	12.5 (10.8-14.0)
Discharge	11.9 (10.2-13.3)	11.2 (9.9-12.8)	9.9 (9.0-11.0)	9.3 (8.5-10.0)	8.8 (8.1-9.6)	10.7 (9.5-12.3)
Discharge hemoglobin MCV, median (IQR), μm^3^^b^	90.0 (86.9-93.3)	90.8 (87.7-94.2)	90.7 (86.7-94.6)	89.5 (84.9-93.7)	86.4 (83.5-90.2)	90.5 (87.1-94.1)
<80	52 (4)	64 (2)	94 (5)	45 (11)	10 (11)	265 (4)
80-100	1110 (92)	3169 (94)	1586 (88)	348 (82)	75 (86)	6288 (91)
>100	45 (4)	141 (4)	131 (7)	29 (7)	2 (2)	348 (5)
RBC						
Any transfusion	218 (18)	585 (17)	645 (36)	279 (66)	68 (78)	1795 (26)
Total units, median (IQR)	0 (0-0)	0 (0-0)	0 (0-2)	2 (0-3)	2 (1-5)	0 (0-1)
Iron therapy	16 (1)	126 (4)	110 (6)	41 (10)	5 (6)	298 (4)
Erythropoiesis-stimulating agents	2 (0.2)	0 (0)	7 (0.4)	7 (1.7)	3 (3.4)	19 (0.3)
Any non-RBC transfusion	126 (10)	438 (13)	341 (19)	99 (23)	23 (26)	1027 (15)

Abbreviations: IQR, interquartile range; MCV, mean corpuscular volume; RBC, allogeneic red blood cell.

SI conversion: To convert hemoglobin to grams per liter, multiply by 10; MCV to femtoliters, multiply by 1.

^a^ Anemia status was defined according to hemoglobin values as nonanemic (≥13.5 g/dL in men; ≥12.0 g/dL in women) and mild (≥10.0 to <13.5 g/dL in men; ≥10.0 to <12.0 g/dL in women), moderate (≥8.0 to <10.0 mg/dL), and severe (<8.0 g/dL) anemia.

^b^ Initial and discharge values over the hospitalization.

### Posthospitalization Anemia

Four hundred forty-one patients (6.4%) died during hospitalization. A total of 62 616 hemoglobin assessments were available for 5373 of the 6460 (83%) critical illness survivors in the first year after hospitalization with a median of 5 (IQR, 2-13) assessments per patient. Patients without follow-up hemoglobin concentration data were generally similar to those with 1 to 5 posthospitalization assessments but were younger (without data: 60.2; IQR, 39.9-76.4 vs with data: 69.5; IQR, 56.3-80.5 years) and had less severe disease as designated by their APACHE III score (without data: 48; IQR, 35-65 vs with data: 61; IQR, 47-75), shorter hospitalizations (without data: 3.8; IQR, 2.1-6.0 vs with data: 5.8; 3.5-9.6 days), and higher hospital discharge hemoglobin concentrations (without data: 11.7; IQR, 10.2-13.3 vs with data: 10.2; IQR, 9.1-11.6 g/dL) than those with more than 5 assessments (eTable 3 in the Supplement). Excluding patients lost to follow-up (n = 158 of 6460 [2.4%]), 86% (5449 of 6302) survived through 12 months. Availability of hemoglobin data was 61% (3660 of 5976) at 3 months, 52% (2974 of 5771) at 6 months, and 47% (2560 of 5449) at 12 months. In complete case analysis, the prevalence of anemia was 56% (95% CI, 55%-58%) at 3 months, 52% (95% CI, 50%-54%) at 6 months, and 45% (95% CI, 43%-47%) at 12 months; temporal trends in hemoglobin concentrations are displayed in eFigure 3 in the Supplement. Estimated anemia prevalence at 3, 6, and 12 months determined through multiple imputation was 52% (95% CI, 50%-53%) at 3 months, 46% (95% CI, 44%-47%) at 6 months, and 41% (95% CI, 40%-43%) at 12 months (eTable 4 in the Supplement).

**Figure 2.**
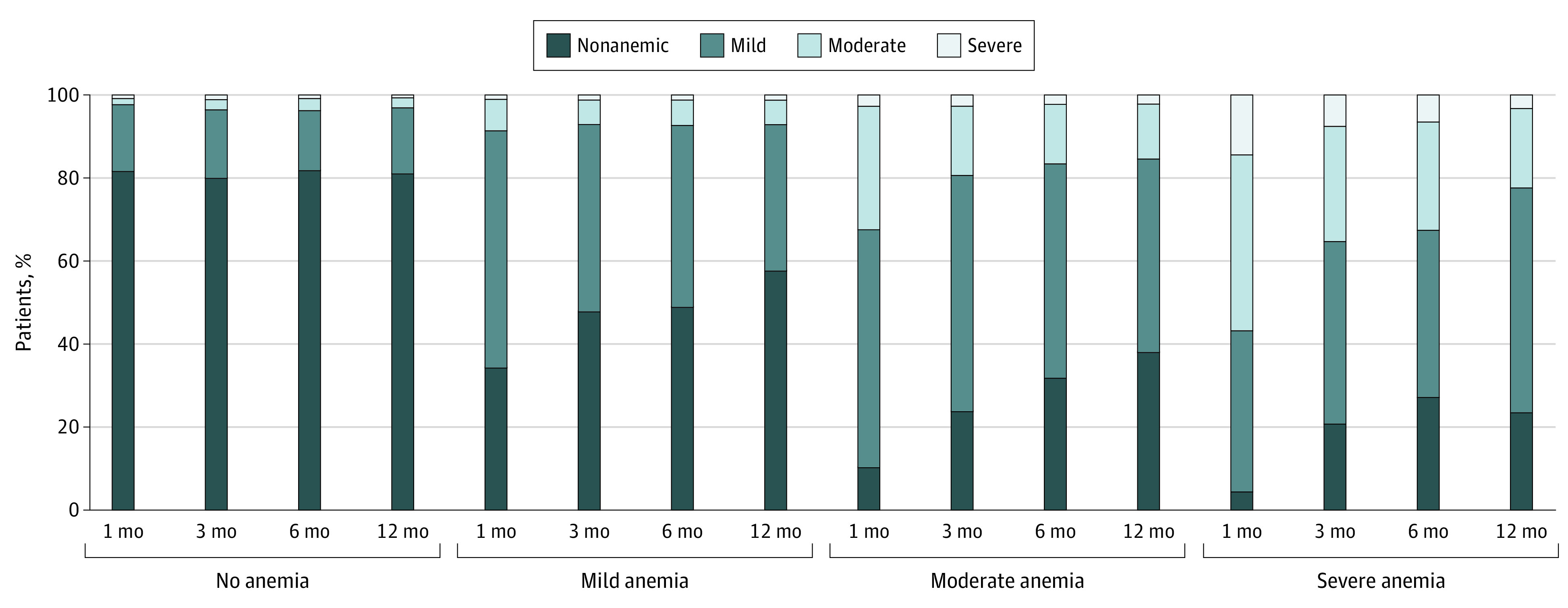
Longitudinal Changes in Anemia Status in the First Year After Hospitalization Stratified by Hospital Discharge Anemia Status

Longitudinal changes in anemia status by hospital discharge anemia status for those with available hemoglobin data are displayed in Figure 2 and eTable 5 in the Supplement. Of those discharged with mild anemia and still under follow-up at 1 year, 88% (2554/2905) survived through 12 months; 51% (95% CI, 49%-54%) remained anemic at 3 months, 50% (95% CI, 48%-53%) at 6 months, and 42% (95% CI, 39%-44%) at 12 months. Eighty-two percent of those with moderate anemia (1612 of 1972) survived through 12 months; 75% (95% CI, 73%-78%) remained anemic at 3 months, 67% (95% CI, 65%-70%) at 6 months, and 61% (95% CI, 58%-64%) at 12 months. Seventy-three percent (129 of 176) of those with severe anemia survived to 12 months; of these, 78% (95% CI, 71%-86%) remained anemic at 3 months, 72% (95% CI, 64%-81%) at 6 months, and 76% (95% CI, 66%-85%) at 12 months. Rates of complete recovery from anemia at 12 months were 58% (95% CI, 56%-61%) for mild anemia, 39% (95% CI, 36%-42%) for moderate anemia, and 24% (95% CI, 15%-34%) for severe anemia. For patients with incident anemia surviving hospitalization, 92% (2120 of 2313) were alive through 12 months. Rates of complete anemia recovery in this group were 56% (95% CI, 53%-64%) at 3 months, 61% (95% CI, 58%-63%) at 6 months, and 69% (95% CI, 66%-72%) at 12 months, with 12-month recovery rates of 73% (95% CI, 69%-76%) for those with mild anemia, 62% (95% CI, 57%-68%) for moderate anemia, and 59% (95% CI, 35%-82%) for severe anemia. Rates of complete recovery for surgical patients discharged with anemia were 40% (95% CI, 38%-43%) at 3 months, 46% (95% CI, 43%-49%) at 6 months, and 56% (95% CI, 53%-59%) at 12 months compared with rates for nonsurgical patients of 35% (95% CI, 32%-37%) at 3 months, 38% (95% CI, 35%-40%) at 6 months, and 44% (95% CI, 41%-47%) at 12 months (eFigure 4 in the Supplement).

Higher discharge hemoglobin concentrations were associated with reduced posthospitalization mortality (HR, 0.95 per 1-g/dL increase; 95% CI, 0.90-0.99; *P* = .02) after multivariable adjustment (complete model summary in eTable 6 in the Supplement). Prehospitalization anemia status was also associated with mortality (*P* < .001, reference nonanemic), including nonavailable hemoglobin data (HR, 0.80; 95% CI, 0.60-1.07), mild anemia (HR, 1.63; 95% CI, 1.37-1.94), moderate anemia (HR, 2.40; 95% CI, 1.90-3.03), and severe anemia (HR, 3.47; 95% CI, 2.33-5.18).

## Discussion

In this large, population-based investigation of critical illness survivors, 41% had anemia before hospital admission, with this number increasing to 80% at hospital discharge. The rate of incident anemia during hospitalization was 74% despite relatively short ICU (median, 1.2 days) and hospital (median, 4.8 days) lengths of stay. At 12 months post hospitalization, nearly half of critical illness survivors with available hemoglobin concentration data remained anemic, including more than one-quarter of survivors without prehospitalization anemia. Higher hospital discharge hemoglobin concentrations were associated with reduced postdischarge mortality.

Beyond this work, there is a paucity of data regarding recovery from anemia in survivors of critical illness. In a prospective investigation of 19 survivors with anemia at ICU discharge, more than 50% experienced persistent anemia at 6 months.^20^ This anemia was accompanied by elevated concentrations of inflammatory markers, including interleukin-6 and C-reactive protein, suggesting the presence of a persistent inflammatory state that may impair erythropoiesis. This inflammatory state has been proposed as a possible factor associated with delayed recovery of physical function after critical illness,^26^ and anemia has also been associated with reduced physical function (ambulatory capacity and activities of daily living) in critical illness survivors.^8^ In noncritically ill older persons, anemia has been associated with a variety of impairments in daily functioning, including decreased mobility,^11,27,28^ muscle weakness,^11,12^ falls,^29^ impaired physical rehabilitation,^30^ reductions in patient-reported physical function,^12^ and global cognitive decline.^31^ With a growing awareness of functional limitations in critical illness survivors,^32^ the potential associations between persistent anemia and functional recovery in this high-risk group should be considered.

### Strengths and Limitations

Strengths of this investigation include a population-based approach with large sample size and longitudinal ascertainment of hemoglobin concentrations before and after critical illness. As such, this work provides a framework for future studies addressing anemia in the critically ill population, including assessment of the associations between hemoglobin recovery and clinical recovery, identification of risk factors for persistent posthospitalization anemia, and evaluations of the safety and efficacy of anemia management interventions on posthospitalization clinical outcomes. Previous investigations not limited to the critically ill have noted associations between hospital-acquired anemia and in-hospital mortality^33^ and 30-day hospital readmission rates.^34^ Building on this information, we found an inverse association between hospital discharge hemoglobin concentrations and mortality after hospitalization, suggesting that anemia attenuation strategies may have important consequences. The prevalence of severe anemia at hospital discharge increased throughout the study period, suggesting increasing tolerance for anemia in clinical practice.

There are limitations of this study. First, we used historical data, and anemia-related diagnostic studies were unavailable for many patients; hence, the causes of anemia were not ascertained. Only 5% of patients received pharmacologic anemia treatment (ie, iron or erythropoiesis-stimulating agents). Second, hemoglobin values were not uniformly available post hospitalization, which may represent random or nonrandom (ie, informative) missingness such that missing data could be indicative of healthier patients not requiring health care contact, those lost to follow-up, or those with significant underlying illness who have died or transitioned to less-aggressive medical care settings. This lack of data impacts our ability to fully assess patterns of anemia recovery, although several approaches were taken to address this issue. Patients with change of residence or death were censored, although it is probable that those who died (14% of hospital survivors) would be less likely to recover from anemia. In addition, clinical characteristics were compared between patients with and without posthospitalization hemoglobin assessments. Those without hemoglobin assessments were generally similar to those with 1 to 5 assessments but were younger and had less severe disease than those with more than 5 assessments, which could lead to underestimation of anemia recovery. Multiple imputation including observed covariates and available hemoglobin assessments was used to impute missing posthospitalization hemoglobin data, with results that were generally consistent with the complete case analysis (45% observed vs 41% estimated anemia prevalence at 12 months). As a third limitation, generalizability to other critically ill populations is unclear. For example, ICU and hospital durations were relatively short in this investigation, which is likely reflective of a propensity to provide intensive care for patients who may be deemed to be lower acuity in other health care and geographic locations. It is possible that anemia recovery may be further impaired in those with longer hospitalizations. In addition, these results were derived from a population that is predominantly of European descent with reliable access to health care, which again may limit generalizability.

## Conclusions

Incident anemia occurs in nearly three-quarters of patients hospitalized for critical illness and often persists through the first year post hospitalization. Further studies are necessary to more fully evaluate associations between posthospitalization anemia and patient-important clinical outcomes. In addition, studies are warranted to distinguish patients likely to recover from anemia after hospitalization from those who may experience prolonged anemia; the latter group may benefit from close outpatient follow-up and/or targeted anemia management strategies.

## Notes

Supplement. **eTable 1.** Data Availability Before Admission **eTable 2.** Hospital Discharge Hemoglobin Concentrations and Anemia Status by Study Year **eTable 3.** Demographic and Clinical Characteristics Based Upon the Number of Hemoglobin Assessments in the First Year After Hospitalization for Critical Illness **eTable 4.** Estimated Proportions of Patients With Anemia Over the First Year of Follow-up According to Anemia Status at Hospital Discharge **eTable 5.** Data Availability at Discharge **eTable 6.** Summary of Adjusted Proportional Hazards Model Assessing the Relationship Between Discharge Hemoglobin and Cumulative Incidence of Mortality Over the 1 Year Following Discharge **eFigure 1.** Study Flow Diagram **eFigure 2.** Changes in Anemia Status During Hospitalization Stratified by Pre-Hospitalization Anemia for Surgical and Non-Surgical Patients **eFigure 3.** Longitudinal Changes in Hemoglobin Concentrations Over the Study Period **eFigure 4.** Longitudinal Changes in Anemia Status in the First Year After Hospitalization Stratified by Hospital Discharge Anemia Status for Surgical and Non-Surgical Patients
