# Supplementary material for: Prevalence of and Recovery From Anemia Following Hospitalization for Critical Illness Among Adults
Source: JAMA Netw Open. 2020 Sep 24;3(9):e2017843. doi: 10.1001/jamanetworkopen.2020.17843 (PMC7516623; doi:10.1001/jamanetworkopen.2020.17843)
Supplement: Supplement. — eTable 1. Data Availability Before Admission eTable 2. Hospital Discharge Hemoglobin Concentrations and Anemia Status by Study Year eTable 3. Demographic and Clinical Characteristics Based Upon the Number of Hemoglobin Assessments in the First Year After Hospitalization for Critical Illness eTable 4. Estimated Proportions of Patients With Anemia Over the First Year of Follow-up According to Anemia Status at Hospital Discharge eTable 5. Data Availability at Discharge eTable 6. Summary of Adjusted Proportional Hazards Model Assessing the Relationship Between Discharge Hemoglobin and Cumulative Incidence of Mortality Over the 1 Year Following Discharge eFigure 1. Study Flow Diagram eFigure 2. Changes in Anemia Status During Hospitalization Stratified by Pre-Hospitalization Anemia for Surgical and Non-Surgical Patients eFigure 3. Longitudinal Changes in Hemoglobin Concentrations Over the Study Period eFigure 4. Longitudinal Changes in Anemia Status in the First Year After Hospitalization Stratified by Hospital Discharge Anemia Status for Surgical and Non-Surgical Patients [file jamanetwopen-e2017843-s001.pdf]

Supplementary Online Content

Warner MA, Hanson AC, Frank RD, et al. Prevalence of and recovery from anemia following hospitalization for critical illness among adults. *JAMA Netw Open*. 2020;3(9):e2017843. doi:10.1001/jamanetworkopen.2020.17843

eTable 1. Data Availability Before Admission

eTable 2. Hospital Discharge Hemoglobin Concentrations and Anemia Status by Study Year

eTable 3. Demographic and Clinical Characteristics Based Upon the Number of Hemoglobin Assessments in the First Year After Hospitalization for Critical Illness

eTable 4. Estimated Proportions of Patients With Anemia Over the First Year of Follow-up According to Anemia Status at Hospital Discharge

eTable 5. Data Availability at Discharge

eTable 6. Summary of Adjusted Proportional Hazards Model Assessing the Relationship Between Discharge Hemoglobin and Cumulative Incidence of Mortality Over the 1 Year Following Discharge

eFigure 1. Study Flow Diagram

eFigure 2. Changes in Anemia Status During Hospitalization Stratified by Pre-Hospitalization Anemia for Surgical and Non-Surgical Patients

eFigure 3. Longitudinal Changes in Hemoglobin Concentrations Over the Study Period

eFigure 4. Longitudinal Changes in Anemia Status in the First Year After Hospitalization Stratified by Hospital Discharge Anemia Status for Surgical and Non-Surgical Patients

This supplementary material has been provided by the authors to give readers additional information about their work.

© 2020 Warner MA et al. *JAMA Network Open.*

eTable 1. Data Availability Before Admission

Variable	Hospital admission, %	ICU admission, %	ICU discharge, %	Hospital discharge, %
**No anemia**				
Available Hb data	100	99.2	96.2	95.0
Death	0	0	2.4	5.0
No available Hb data	0	0.8	1.4	0
**Mild anemia**				
Available Hb data	100	99.2	93.6	90.7
Death	0	0	5.1	9.3
No available Hb data	0	0.8	1.3	0
**Moderate anemia**				
Available Hb data	100	99.1	93.4	88.9
Death	0	0	5.5	11.1
No available Hb data	0	0.9	1.2	0
**Severe anemia**				
Available Hb data	100	100	95.4	94.3
Death	0	0	4.6	5.7
No available Hb data	0	0	0	0

Hb, hemoglobin; ICU, intensive care unit.

© 2020 Warner MA et al. *JAMA Network Open.*

**eTable 2**. Hospital discharge hemoglobin concentrations and anemia status by study year.				
		Sex		
		Male	Female	Total
2010		(N=617)	(N=530)	(N=1147)
	**Hemoglobin (g/dL)**	11.2 (9.9, 13.0)	10.5 (9.6, 11.7)	10.8 (9.7, 12.4)
	**Anemia at hospital discharge**, n (%)			
	Non-anemic	119 (19.3%)	107 (20.2%)	226 (19.7%)
	Mild	332 (53.8%)	246 (46.4%)	578 (50.4%)
	Moderate	160 (25.9%)	172 (32.5%)	332 (28.9%)
	Severe	6 (1.0%)	5 (0.9%)	11 (1.0%)
2011		(N=502)	(N=430)	(N=932)
	**Hemoglobin (g/dL)**	11.2 (9.8, 12.8)	10.5 (9.5, 11.6)	10.8 (9.6, 12.2)
	**Anemia at hospital discharge**, n (%)			
	Non-anemic	82 (16.3%)	82 (19.1%)	164 (17.6%)
	Mild	274 (54.6%)	191 (44.4%)	465 (49.9%)
	Moderate	144 (28.7%)	151 (35.1%)	295 (31.7%)
	Severe	2 (0.4%)	6 (1.4%)	8 (0.9%)
2012		(N=540)	(N=467)	(N=1007)
	**Hemoglobin (g/dL)**	11.2 (9.6, 13.0)	10.3 (9.3, 11.5)	10.7 (9.4, 12.4)
	**Anemia at hospital discharge**, n (%)			
	Non-anemic	100 (18.5%)	88 (18.8%)	188 (18.7%)
	Mild	269 (49.8%)	189 (40.5%)	458 (45.5%)
	Moderate	159 (29.4%)	179 (38.3%)	338 (33.6%)
	Severe	12 (2.2%)	11 (2.4%)	23 (2.3%)
2013		(N=488)	(N=389)	(N=877)
	**Hemoglobin (g/dL)**	11.2 (9.5, 12.9)	10.5 (9.4, 11.7)	10.8 (9.4, 12.4)
	**Anemia at hospital discharge**, n (%)			
	Non-anemic	91 (18.6%)	79 (20.3%)	170 (19.4%)
	Mild	238 (48.8%)	167 (42.9%)	405 (46.2%)
	Moderate	147 (30.1%)	127 (32.6%)	274 (31.2%)
	Severe	12 (2.5%)	16 (4.1%)	28 (3.2%)
2014		(N=488)	(N=376)	(N=864)
	**Hemoglobin (g/dL)**	11.3 (9.6, 13.1)	10.5 (9.3, 11.9)	10.8 (9.5, 12.5)
	**Anemia at hospital discharge**, n (%)			
	Non-anemic	94 (19.3%)	92 (24.5%)	186 (21.5%)
	Mild	244 (50.0%)	138 (36.7%)	382 (44.2%)
	Moderate	131 (26.8%)	138 (36.7%)	269 (31.1%)
	Severe	19 (3.9%)	8 (2.1%)	27 (3.1%)
2015		(N=453)	(N=341)	(N=794)
	**Hemoglobin (g/dL)**	11.4 (9.5, 13.1)	10.3 (9.2, 12.0)	11.0 (9.3, 12.5)
	**Anemia at hospital discharge**, n (%)			
	Non-anemic	96 (21.2%)	88 (25.8%)	184 (23.2%)
	Mild	212 (46.8%)	105 (30.8%)	317 (39.9%)
	Moderate	120 (26.5%)	133 (39.0%)	253 (31.9%)
	Severe	25 (5.5%)	15 (4.4%)	40 (5.0%)
2016		(N=480)	(N=359)	(N=839)
	**Hemoglobin (g/dL)**	11.3 (9.6, 13.0)	10.3 (9.2, 11.7)	10.7 (9.4, 12.4)
	**Anemia at hospital discharge**, n (%)			
	Non-anemic	84 (17.5%)	76 (21.2%)	160 (19.1%)
	Mild	250 (52.1%)	125 (34.8%)	375 (44.7%)
	Moderate	123 (25.6%)	137 (38.2%)	260 (31.0%)
	Severe	23 (4.8%)	21 (5.8%)	44 (5.2%)
Hemoglobin provided as median (IQR).				

© 2020 Warner MA et al. *JAMA Network Open.*

eTable 3. Demographic and clinical characteristics based upon the number of hemoglobin assessments in the first year after hospitalization for critical illness

	**None** (N=1087)	**1-5** (N=2830)	**6 or more** (N=2543)	**Total** (N=6460)
**Age (years)**	60.2 (39.9, 76.4)	64.6 (50.3, 78.2)	69.5 (56.3, 80.5)	66.3 (51.5, 78.9)
**Charlson comorbidity index**	1 (0, 2)	1 (0, 3)	3 (1, 5)	2 (0, 4)
**APACHE III score**	48 (35, 65)	52 (39, 67)	61 (47, 75)	55 (41, 70)
**Hospital admission Hb (g/dL)**	13.4 (11.9, 14.6)	13.0 (11.3, 14.2)	11.8 (10.1, 13.3)	12.6 (10.9, 14.0)
**Hospital discharge Hb (g/dL)**	11.7 (10.2, 13.3)	11.1 (9.7, 12.6)	10.2 (9.1, 11.6)	10.8 (9.5, 12.4)
**Hospital length of stay (days)**	3.8 (2.1, 6.0)	4.4 (2.7, 6.8)	5.8 (3.5, 9.6)	4.8 (2.9, 7.7)
**Sex**, n (%)				
Male	647 (59.5%)	1556 (55.0%)	1365 (53.7%)	3568 (55.2%)
Female	440 (40.5%)	1274 (45.0%)	1178 (46.3%)	2892 (44.8%)
**Pre-hospitalization anemia**, n (%)				
Lost to follow-up	49 (4.5%)	56 (2.0%)	25 (1.0%)	130 (2.0%)
Not available	310 (28.5%)	526 (18.6%)	191 (7.5%)	1027 (15.9%)
Non-anemic	508 (46.7%)	1589 (56.1%)	1107 (43.5%)	3204 (49.6%)
Mild	169 (15.5%)	566 (20.0%)	907 (35.7%)	1642 (25.4%)
Moderate	43 (4.0%)	73 (2.6%)	259 (10.2%)	375 (5.8%)
Severe	8 (0.7%)	20 (0.7%)	54 (2.1%)	82 (1.3%)
**Admission type**, n (%)				
Medical	691 (63.6%)	1625 (57.4%)	1578 (62.1%)	3894 (60.3%)
Surgical	396 (36.4%)	1205 (42.6%)	965 (37.9%)	2566 (39.7%)

Hb – hemoglobin. APACHE– Acute Physiology and Chronic Health Evaluation.
Presented as median (IQR) for continuous data and n (%) for categorical data.

© 2020 Warner MA et al. *JAMA Network Open.*

**eTable 4**. Estimated proportions of patients with anemia over the first year of follow-up according to anemia status at hospital discharge*			
Anemia status at hospital discharge/time-point	*Proportion anemic (95% CI)*	*Proportion anemic – Medical (95% CI)*	*Proportion anemic – Surgical (95% CI)*
Overall			
1 month	0.62 (0.61, 0.63)	0.57 (0.55, 0.58)	0.70 (0.68, 0.72)
3 months	0.52 (0.50, 0.53)	0.49 (0.47, 0.51)	0.55 (0.52, 0.57)
6 months	0.46 (0.44, 0.47)	0.45 (0.43, 0.47)	0.46 (0.44, 0.49)
12 months	0.41 (0.40, 0.43)	0.43 (0.41, 0.45)	0.39 (0.37, 0.42)
Non-anemic			
1 month	0.17 (0.15, 0.20)	0.17 (0.14, 0.20)	0.19 (0.13, 0.26)
3 months	0.20 (0.17, 0.22)	0.19 (0.17, 0.22)	0.20 (0.13, 0.26)
6 months	0.18 (0.16, 0.21)	0.19 (0.15, 0.22)	0.17 (0.11, 0.24)
12 months	0.19 (0.16, 0.22)	0.20 (0.16, 0.23)	0.16 (0.10, 0.23)
Mild			
1 month	0.62 (0.60, 0.64)	0.61 (0.58, 0.63)	0.64 (0.61, 0.67)
3 months	0.50 (0.47, 0.52)	0.51 (0.48, 0.54)	0.47 (0.44, 0.51)
6 months	0.45 (0.42, 0.47)	0.47 (0.44, 0.50)	0.41 (0.38, 0.44)
12 months	0.40 (0.38, 0.42)	0.44 (0.41, 0.47)	0.35 (0.31, 0.38)
Moderate			
1 month	0.88 (0.86, 0.90)	0.91 (0.89, 0.93)	0.86 (0.83, 0.88)
3 months	0.74 (0.72, 0.76)	0.80 (0.77, 0.83)	0.69 (0.66, 0.72)
6 months	0.63 (0.61, 0.66)	0.72 (0.68, 0.76)	0.57 (0.53, 0.60)
12 months	0.57 (0.54, 0.60)	0.68 (0.63, 0.72)	0.49 (0.45, 0.53)
Severe			
1 month	0.95 (0.92, 0.98)	0.97 (0.93, 1.00)	0.93 (0.87, 0.99)
3 months	0.80 (0.73, 0.87)	0.86 (0.78, 0.94)	0.73 (0.61, 0.84)
6 months	0.71 (0.64, 0.79)	0.80 (0.71, 0.90)	0.62 (0.49, 0.74)
12 months	0.70 (0.61, 0.78)	0.82 (0.72, 0.93)	0.57 (0.44, 0.70)

© 2020 Warner MA et al. *JAMA Network Open.*

eTable 4. Estimated proportions of patients with anemia over the first year of follow-up according to anemia status at hospital discharge*

Anemia status at hospital discharge/time-point	*Proportion anemic (95% CI)*	*Proportion anemic – Medical (95% CI)*	*Proportion anemic – Surgical (95% CI)*

* Proportions are estimated from those alive at the given time-point (i.e. patients known to be deceased do not have imputed values after month of death). Imputation was done on the raw hemoglobin values and converted to anemic yes/no using the criteria previously described. Fifty imputed datasets were generated using hospitalization covariates and outcomes to inform imputation. Variables in the imputation model included age, hospital length of stay, surgical/medical admission, sex, APACHE 3 score, log follow-up time, last follow-up status (alive/dead), any RBC transfusion, any non-RBC transfusion, hospital admission, ICU admission, and hospital discharge hemoglobin values, 1-, 3-, 6-, 9-, and 12-month hemoglobin values.

© 2020 Warner MA et al. *JAMA Network Open.*

eTable 5. Data Availability at Discharge

Variable	1 Month, %	3 Month, %	6 Month, %	12 Month, %
**No anemia**				
Available Hb data	49.7	43.6	36.0	33.3
Death	2.8	3.8	5.2	7.4
Follow-up	0.2	0.5	0.7	2.3
No available Hb data	47.3	52.0	58.1	57.0
**Mild anemia**				
Available Hb data	65.2	55.5	46.0	40.6
Death	3.2	5.6	8.3	11.8
Follow-up	0.3	0.7	1.2	2.5
No available Hb data	31.3	38.2	44.5	45.1
**Moderate anemia**				
Available Hb data	77.3	65.3	51.5	42.1
Death	5.3	9.6	13.4	17.8
Follow-up	0.3	0.8	1.1	2.4
No available Hb data	17.0	24.2	34.0	37.7
**Severe anemia**				
Available Hb data	84.5	71.8	57.5	40.9
Death	7.7	13.8	18.8	26.0
Follow-up	0.6	1.1	1.7	2.8
No available Hb data	7.2	13.3	22.1	30.4

Hb, hemoglobin; ICU, intensive care unit.

© 2020 Warner MA et al. *JAMA Network Open.*

eTable 6. Summary of adjusted proportional hazards model assessing the relationship between discharge hemoglobin and cumulative incidence of mortality over the 1 year following discharge*

Characteristic	*Hazard Ratio*	*95% Confidence Interval*	*P-value*
Age, per 10 years	1.36	(1.29 to 1.43)	<.001
Type of admission			<.001
Surgical (n=2566)	Referent		
Medical (n=3894)	2.05	(1.75 to 2.40)	
Sex			0.45
Female (n=2892)	Referent		
Male (n=3568)	1.05	(0.92 to 1.21)	
Charlson comorbidity index, per 1 point	1.10	(1.07 to 1.12)	<.001
APACHE 3 score, per 10 points	1.15	(1.11 to 1.18)	<.001
ICU length of stay, per 1 day	1.03	(1.01 to 1.05)	0.007
Pre-hospitalization level of anemia			<.001
None (n=3204)	Referent		
Not available (n=1157)	0.80	(0.60 to 1.07)	
Mild (n=1642)	1.63	(1.37 to 1.94)	
Moderate (n=375)	2.40	(1.90 to 3.03)	
Severe (n=82)	3.47	(2.33 to 5.18)	
Discharge Hemoglobin, per g/dL	0.95	(0.90 to 0.99)	0.02

* Hazard ratios represent the increased hazard of event associated with the given covariate. Patients who died in hospital were excluded from the analysis (n=441/6901, 6.4%).

© 2020 Warner MA et al. *JAMA Network Open.*

eFigure 1. Study flow diagram

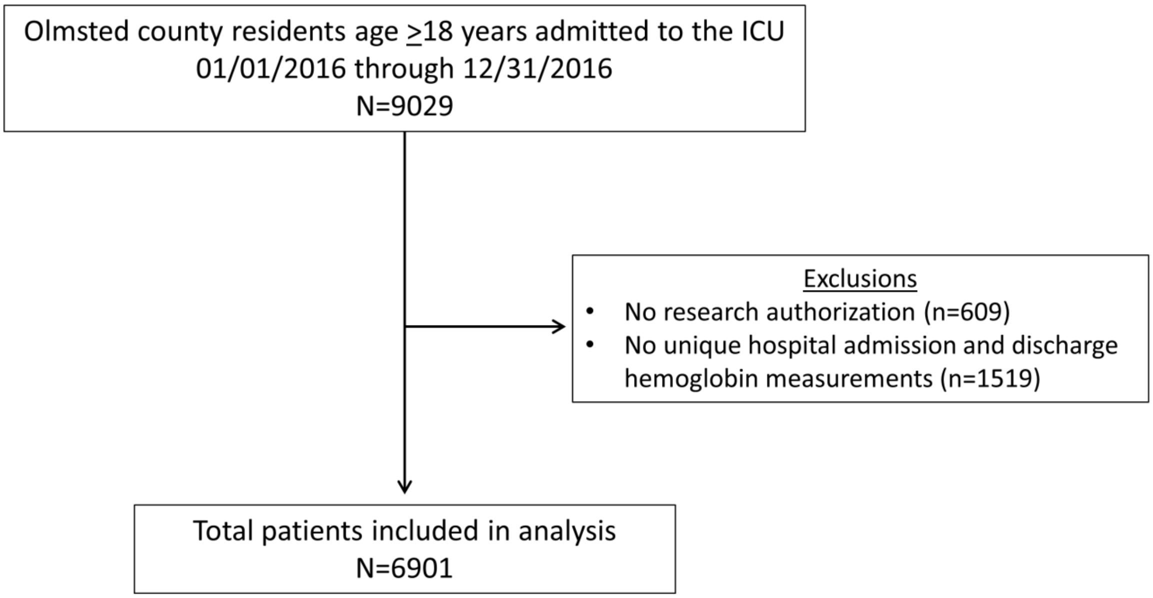

© 2020 Warner MA et al. *JAMA Network Open.*

eFigure 2. Changes in anemia status during hospitalization stratified by pre-hospitalization anemia for surgical and non-surgical patients.

Non-Surgical **Surgical**

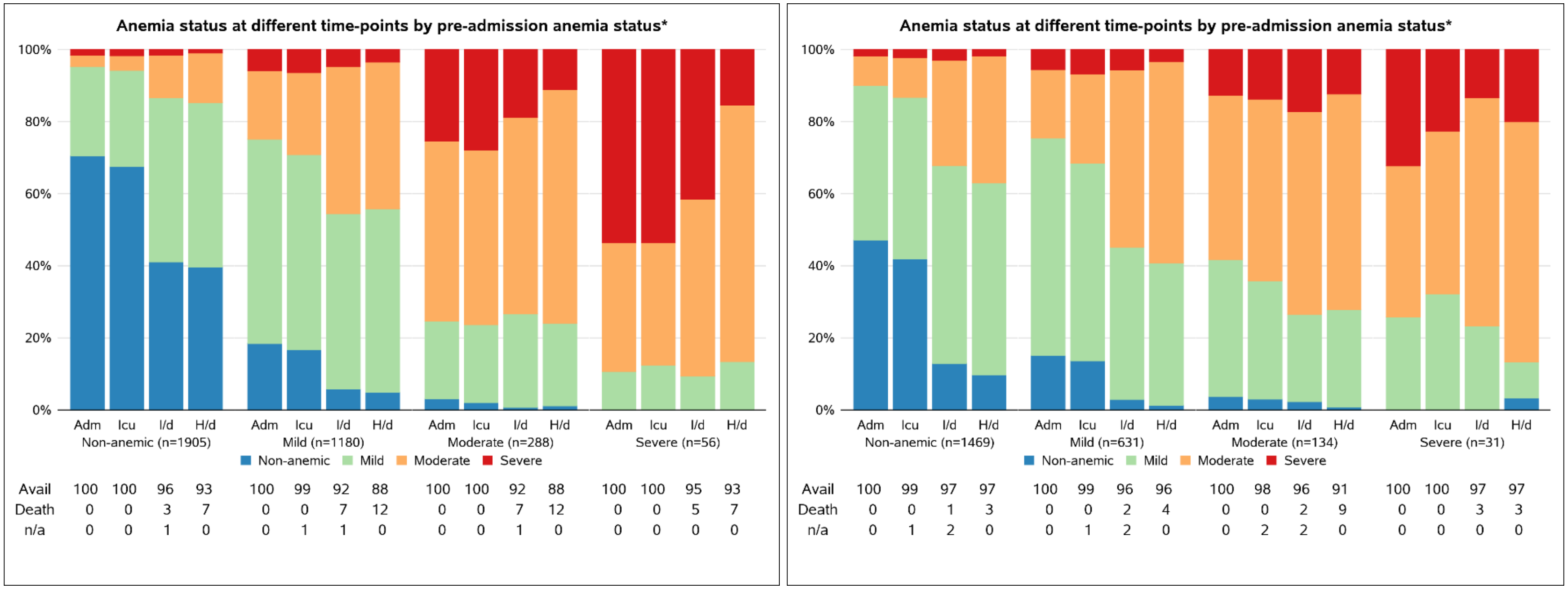

© 2020 Warner MA et al. *JAMA Network Open.*

eFigure 3. Longitudinal changes in hemoglobin concentrations over the study period.

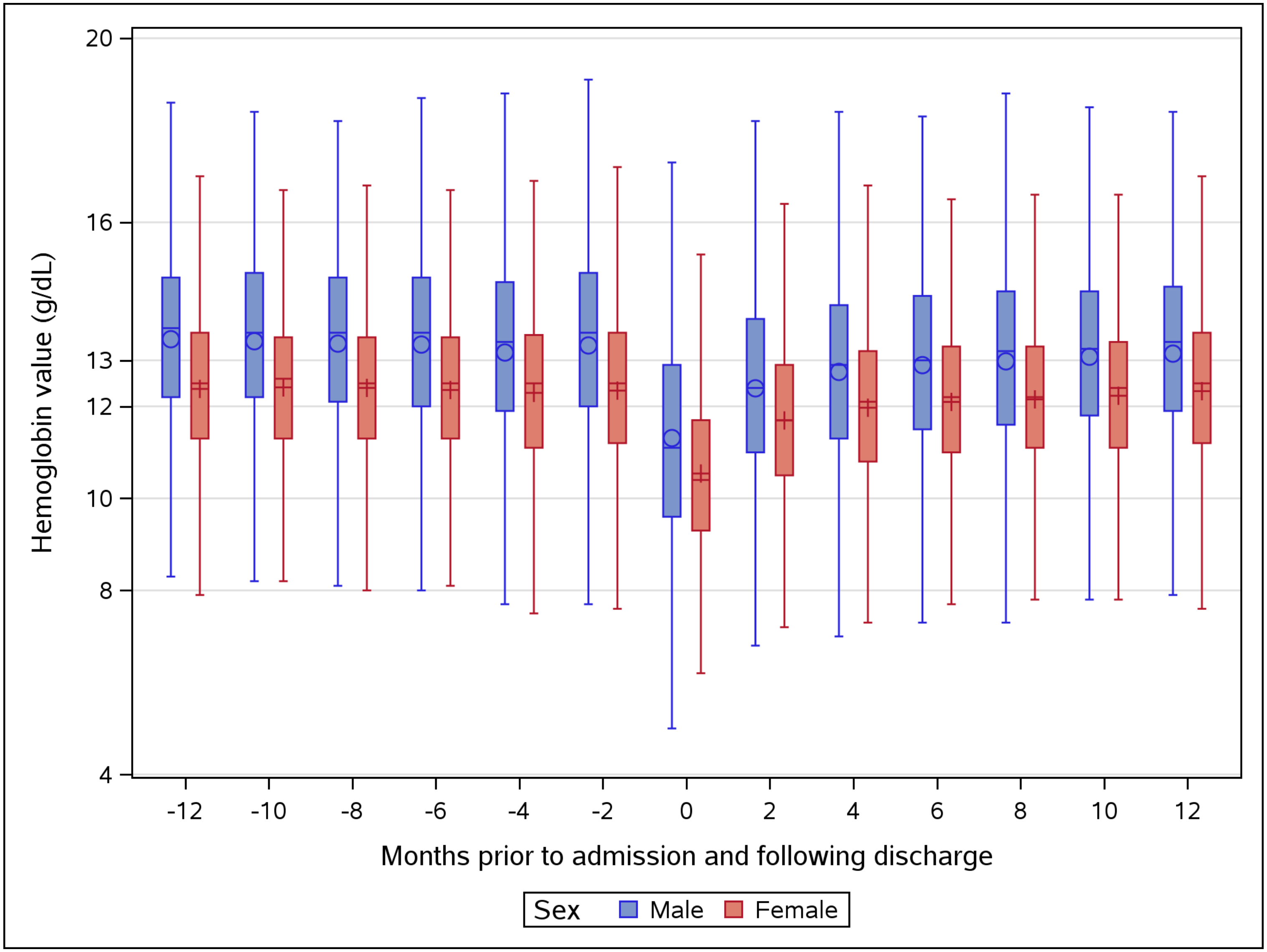

© 2020 Warner MA et al. *JAMA Network Open.*

eFigure 4. Longitudinal changes in anemia status in the first year after hospitalization stratified by hospital discharge anemia status for surgical and non-surgical patients

Non-Surgical

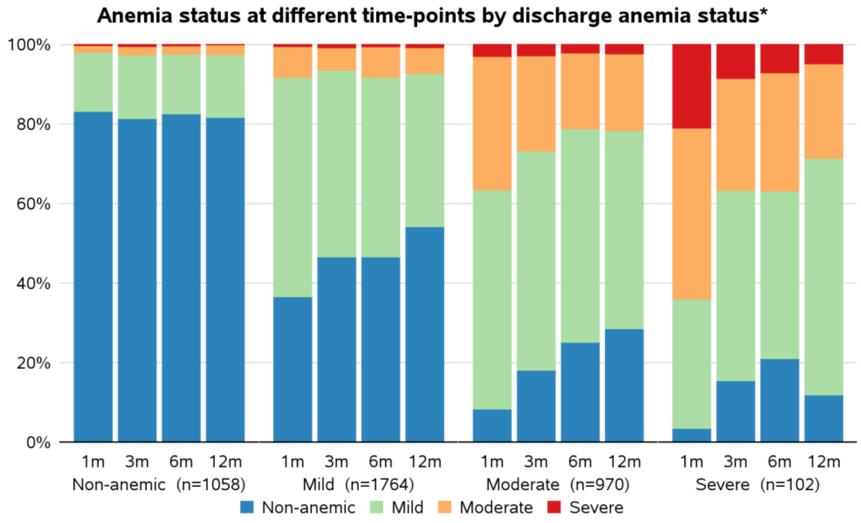

	Non-anemic 1m	3m	6m	12m	Mild 1m	3m	6m	12m	Moderate 1m	3m	6m	12m	Severe 1m	3m	6m	12m
Avail	50.8	45.4	37.1	34.7	65.5	55.7	46.5	41.3	77.7	63.7	50.8	41.1	84.3	69.6	55.9	41.2
Death	2.7	3.8	5.1	7.4	4.5	7.7	11.4	15.3	8.4	15.2	20.2	26.1	8.8	17.6	24.5	33.3
f/u	0.0	0.4	0.6	2.0	0.2	0.5	1.0	2.6	0.3	0.9	1.1	2.8	1.0	2.0	2.0	2.9
n/a	46.5	50.5	57.3	56.0	29.8	36.2	41.0	40.8	13.6	20.2	27.8	30.0	5.9	10.8	17.6	22.5

Surgical

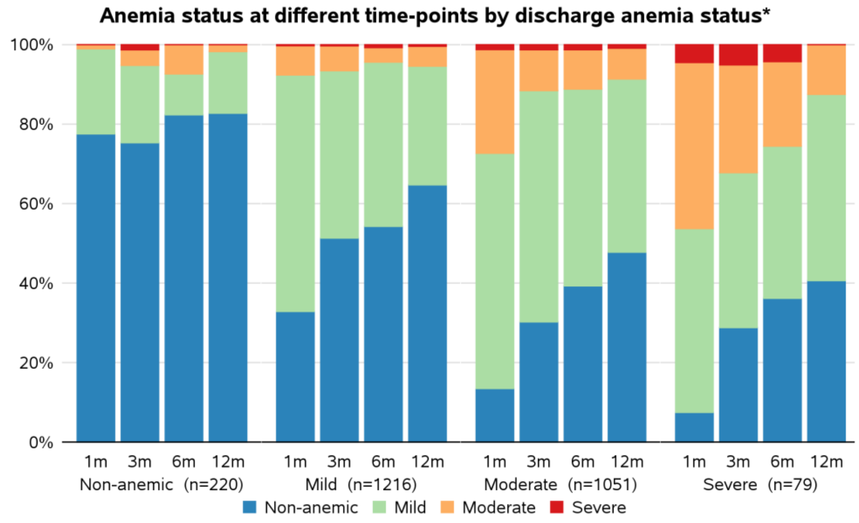

	Non-anemic 1m	3m	6m	12m	Mild 1m	3m	6m	12m	Moderate 1m	3m	6m	12m	Severe 1m	3m	6m	12m
Avail	44.5	35.0	30.9	26.4	64.8	55.3	45.1	39.6	77.0	66.7	52.0	43.0	84.8	74.7	59.5	40.5
Death	3.2	4.1	5.5	7.7	1.4	2.7	3.9	6.7	2.5	4.6	7.0	10.2	6.3	8.9	11.4	16.5
f/u	0.9	1.4	1.4	3.6	0.4	1.1	1.5	2.4	0.4	0.8	1.1	2.1	0.0	0.0	1.3	2.5
n/a	51.4	59.5	62.3	62.3	33.4	41.0	49.5	51.3	20.2	28.0	39.8	44.7	8.9	16.5	27.8	40.5

© 2020 Warner MA et al. *JAMA Network Open.*
